# 质谱成像技术及其在乳腺癌研究中的应用

**DOI:** 10.3724/SP.J.1123.2020.10005

**Published:** 2021-06-08

**Authors:** Mengting ZHANG, Yulu ZHANG, Haojiang WANG, Ning LI, Bo LI, Hong XIAO, Wei BIAN, Zongwei CAI

**Affiliations:** 1.山西医科大学基础医学院生物化学与分子生物学教研室, 山西 太原 030001; 1. Department of Biochemistry and Molecular Biology, School of Basic Medical Science, Shanxi Medical University, Taiyuan 030001, China; 2.山西医科大学第一医院病理科, 山西 太原 030001; 2. Department of Pathology, First Hospital of Shanxi Medical University, Taiyuan 030001, China; 3.香港浸会大学化学系环境与生物分析国家重点实验室, 香港 999077; 3. State Key Laboratory of Environmental and Biological Analysis, Department of Chemistry, Hong Kong Baptist University, Hong Kong 999077, China

**Keywords:** 质谱成像技术, 样品前处理, 乳腺癌, mass spectrometry imaging technology, sample preparation, breast cancer

## Abstract

乳腺癌是女性最常见的恶性肿瘤,其发病率在世界范围内呈现上升趋势,是威胁女性健康的重要疾病之一。随着现代医学技术的快速发展,早期有效的诊断和筛查方法能够改善乳腺癌患者生存率和提高其生活质量。由于乳腺癌肿瘤具有非常显著的异质性,这对于诊断和筛查带来了较大困难,亟须在肿瘤演进时间信息中,继续引入生物分子的空间信息,从而对其异质性、肿瘤微环境等进行准确的追踪。质谱成像技术,可在免标记的前提下利用离子质荷比的特性发现生物组织中的各种分子,并研究这些分子的时间和空间信息,对其进行准确的定性、定量和空间定位。目前,通过质谱成像技术可直接获取药物及其代谢物、内源性代谢物、脂质、多肽和蛋白质等在组织中的空间分布信息,为肿瘤分子分型诊断和确认以及相关抗肿瘤药物的筛选提供了新的思路和研究方向。该综述以乳腺癌相关的生物样品制备和研究进展为主要内容,从小分子样本、大分子样本、石蜡包埋样本、基质喷涂方式、常用离子源等方面阐述质谱成像中样本制备的重要性以及样品制备过程中存在的难点问题。同时,以细胞模型、动物模型和临床肿瘤标本为研究对象,汇总了质谱成像技术在乳腺癌方面的应用进展,并进行了展望,为开展癌症精准分型研究和药物药效的快速筛查提供了重要依据。

## Abstract (en)

The incidence of breast cancer, one of the most common malignancies affecting women, is increasing significantly worldwide. Given the rapid development of medical technology, early and effective diagnostic methods should be able to improve the survival rate and quality of life of patients suffering from disease. However, although existing treatment options, including chemotherapy and endocrine therapies, have greatly improved the survival of patients, disease recurrence in the long term remains a challenge. Because breast cancer is a heterogeneous and complex disease, which includes several subtypes with different responses to treatment, the continual acquisition of spatial information on related biomolecules is important for accurate tracking of the tumor heterogeneity and microenvironment. At present, prognostic and predictive biomarkers, such as human epidermal growth factor receptor 2 (HER2), estrogen receptor (ER), Ki-67, progesterone receptor (PR), and programmed death-ligand 1 (PD-L1), are validated for use in the decision-making over breast cancer therapies. Mass spectrometry imaging (MSI) is a useful technique for acquiring molecular information about biological tissues, including qualitative, quantitative, and spatial distribution information, because it is based on the ion mass-to-charge ratio of the biomolecules and avoids the need for their labeling and staining. MSI can also acquire molecular information on drugs and their metabolites, as well as that on molecules related to endogenous metabolism, such as lipids, peptides, and proteins. Of the various ion sources available for MSI, the most popular are matrix-assisted laser desorption ionization, secondary ion mass spectrometry, and desorption electrospray ionization, and modifications or derivatives of these sources are still emerging. MSI-based techniques provide new ideas and directions for the molecular typing of tumors, as well as knowledge on the metabolism of related antitumor drugs.

The process of MSI analysis generally involves tissue acquisition, section preparation, mass spectrometry ionization, map acquisition, and data analysis, with the most crucial step being sample handling to preserve the original chemical and location information of the analytes. The sample preparation steps are sample collection, storage, and slicing, tissue pretreatment, and matrix spraying. This review focuses mainly on the preparation of biological specimens for MSI analysis and the recent progress made in breast cancer research with this technology. With regard to sample preparation, four aspects are discussed: small-molecule samples, macromolecular samples, paraffin-embedded samples, and matrix spraying methods. To solve the difficulties associated with small-molecule sample processing, including the low extraction efficiency for certain lipids and matrix interference in the low-molecular-weight region, the addition of a cationic reagent to the extractant, the use of a new matrix, and tissue derivatization have been used. In the review of macromolecular sample processing, several different washing protocols are summarized. With regard to paraffin-embedded samples, the solutions to several common problems are reviewed. Additionally, the application of MSI to three models associated with breast cancer research is discussed, viz. cell models, animal models, and clinical tumor samples. For these models, MSI technology is used to evaluate the penetration and metabolism of antitumor agents in breast cancer, which can better reflect the malignant transformation of cells and changes in the microenvironment. With regard to lipid molecules, the use of MSI to study differences in their spatial distribution may provide a better understanding of the relationship between lipid metabolism and cancer. This review also provides important information for accurate molecular typing and drug screening in cancer research. Analytically, the tissue preparation method, tissue storage conditions, instrumentation choice, and experimental parameters have all been associated with variability in the imaging and mass-spectral qualities of MSI, thereby affecting the performance of the method. Large-scale studies using diverse sample cohorts are therefore needed to properly evaluate the robustness of MSI molecular markers and workflows for the clinical diagnosis and characterization of breast cancer variants. Our review provides strong evidence that MSI is a reliable, highly reproducible, and rapid technique for the diagnosis of breast cancer biopsies and may be useful in clinical application.

乳腺癌是导致妇女因癌症死亡的主要原因之一,它是一种高度异质性疾病,包括几种亚型^[1]^。目前,早期乳腺癌的死亡率已大大降低,远低于40年前的水平,治愈率为90%,但是面对晚期乳腺癌的治疗和治愈后的复发仍然存在挑战^[2]^。基于分子亚型的不同,乳腺癌表现出不同的基因表达模式,主要由雌激素受体、孕激素受体和人表皮生长因子受体-2的状态表达情况决定^[3]^。乳腺癌的早期诊治中需要更精确地评估敏感的生物标记物,在分子水平上对乳腺癌进行精确的诊断和分型,提高乳腺癌的诊断和预后情况,指导分子靶向治疗。

质谱成像(mass spectrometry imaging, MSI)作为一种免标记的分子成像技术,可直接获取药物及其代谢物以及脂质、多肽、蛋白质等内源性代谢物在组织中的分子信息和空间分布信息,在生物、医学等领域表现出良好的应用前景。基于电离方式不同,常见的质谱成像技术可分为基质辅助激光解吸电离质谱成像(matrix-assisted laser desorption/ionization mass spectrometry imaging, MALDI-MSI)^[4]^,解吸电喷雾离子化质谱成像(desorption electrospray ionization mass spectrometry imaging, DESI-MSI)^[5]^和二次离子质谱成像(secondary ion mass spectrometry mass spectrometry imaging, SIMS-MSI)^[6]^。

质谱成像分析的一般流程为组织获取、切片制备、质谱电离、图谱获取和数据分析。样品前处理是影响MSI结果的关键步骤,处理方法与待测物性质和样品类型密切相关,正确的样品制备可以保持分子的来源、分布和丰度,获得高质量的信号和足够的空间分辨率^[7]^。在MALDI-MSI中,基质的选择和喷涂方式同样决定了成像分析的结果。本综述介绍了质谱成像技术中对于不同类型样品的处理及其在乳腺癌不同研究模型的应用进展,为开展乳腺癌的精准诊断和分型提供依据。

## 1 MSI样品处理

MSI样品处理的方法与样品类型、待测物自身的性质相关,基本过程包括收集样品、储存、切片、组织预处理、基质喷涂等方面。样品制备对于实验结果的可靠性和可重复性至关重要,显著影响MSI的分辨率、灵敏度和数据的准确性。

### 1.1 小分子

内源性代谢物,包括低相对分子质量代谢物(*m/z* <500)和脂质,是细胞结构的重要组成部分,它们在细胞信号传导,氧化应激反应和能量代谢中起着至关重要的作用。小分子代谢物的成像可以采用DESI、nano-DESI、MALDI等离子源或者不同的电离方式(见表1)。使用DESI和nano-DESI对生物样品进行成像时只需要很少的样品预处理步骤甚至不需要进行处理。Nguyen等^[13]^使用甲醇和水组成的常用脂质萃取溶剂对小鼠肺部组织中的脂质进行了nano-DESI-MSI分析,实现了对小鼠肺组织中20个脂质亚类中的265种独特脂质和19种代谢物(共284种)的鉴定,覆盖率达到40%。实验中未检测到的脂质可能是由于该类脂质的含量低、萃取剂对其萃取效率低或脂质的离子化程度低。在萃取剂中添加阳离子试剂可以增强特定脂质的离子化程度,例如Duncan等^[8]^在萃取溶剂中添加Ag^+^,实现了对含量较低的前列腺素的nano-DESI-MSI分析。组织基质中的离子抑制效应对药物的质谱成像有较大的影响,是MSI在临床药物研究中所面临的难点和重点问题。Song等^[14]^开发了一种原位水凝胶调节方法,来增强空气辅助解吸电喷雾电离质谱成像(Air-flow-assisted desorption electrospray ionization-mass spectrometry imaging, AFADESI-MSI)的灵敏度。该方法使用固相水凝胶“冲洗”组织切片代替在溶剂中对其进行冲洗或消化处理。并检验了此方法对各种不同理化性质药物(如紫杉醇、罗红霉素、利血平、双氯芬酸等)的适用性。结果表明,该方法可以显著降低水溶性季铵盐和无机盐对离子的抑制作用,将药物信号增强2到25倍。

**表 1:** 不同电离方式在检测小分子时的难点及解决方法

Ionization methods	Advantages	Difficulties	Solutions	Refs.
DESI & nano-DESI	requires less sample pretreatment, no matrix, high resolution of nano-DESI	low extraction efficiency for certain lipids	add cationic reagent to extractant	[8]
MALDI	need matrix, can realize the analysis from lipids and other small biological molecules to proteins and other biological macromolecules, high resolution	matrix interference in the low molecular weight region	new matrix: BNDM, Gly-3AQ	[9,10]
		carbon-carbon double bond positional isomer	Paternò-Büchi reaction	[11]
		detection of certain specific lipids	on situ tissue derivatization	[12]

DESI: desorption electrospray ionization; MALDI: matrix-assisted laser desorption/ionization; BNDM: 1,1'-binaphthyl-2,2'-diamine; Gly-3AQ: glycosyl-3-aminoquinoline.

使用MALDI-MSI对小分子进行成像时,会存在多种与基质相关的离子信号,干扰成像的结果和质量。为了解决这个问题,研究人员找到了新的基质用于MALDI-MSI(见表2)。Sun等^[9]^应用背景干扰小、灵敏度高、可同时应用于正离子和负离子模式检测的1,1'-联萘-2,2'-二胺(BNDM)作为基质,实现了对大鼠大脑中氨基酸、有机酸、核苷、核苷酸、含氮碱基、胆固醇、多肽、脂肪酸、磷脂酰乙醇胺等301个负代谢物离子和胆碱、肉碱、多胺、肌酸、磷脂酰胆碱等175个正代谢物离子的成像,通过探索整体脂质的变化来发现与病理状态相关的功能分子。图1为用BNDM作为MALDI-MSI的新基质检测大鼠大脑中代谢物的示意图。Guan等^[15]^使用聚乙烯吡咯烷酮包被的银纳米颗粒(AgNPs)作为MALDI-MSI的基质,实现了对脑组织中脂肪酸、甘油酯、甘油磷脂、鞘脂和固醇的同时成像。

**表 2:** 新基质及其优势

Matrix	Advantages	Refs.
BNDM & Gly-3AQ	overcome background interference in the low-quality range, and enhance the detection intensity of small molecule metabolites in the range of m/z<500; BNDM can be used for positive and negative ion mode detection; Gly-3AQ has acid response, and the optimum pH range is 2-7.	[9,10]
Polyvinylpyrrolidone capped silver nanoparticles (AgNPs@PVP)	enhanced comprehensive imaging of lipids	[15]
Polydopamine-capped AgNPs (AgNPs@PDA)	decrease the strength of phosphatidylcholine, and increase the strength of glycerophospholipid and sphingomyelin	[16]
Combination of sodium doping and 2,5-dihydrobenzoic acid	detection of neutral lipids	[17]

**图 1:**
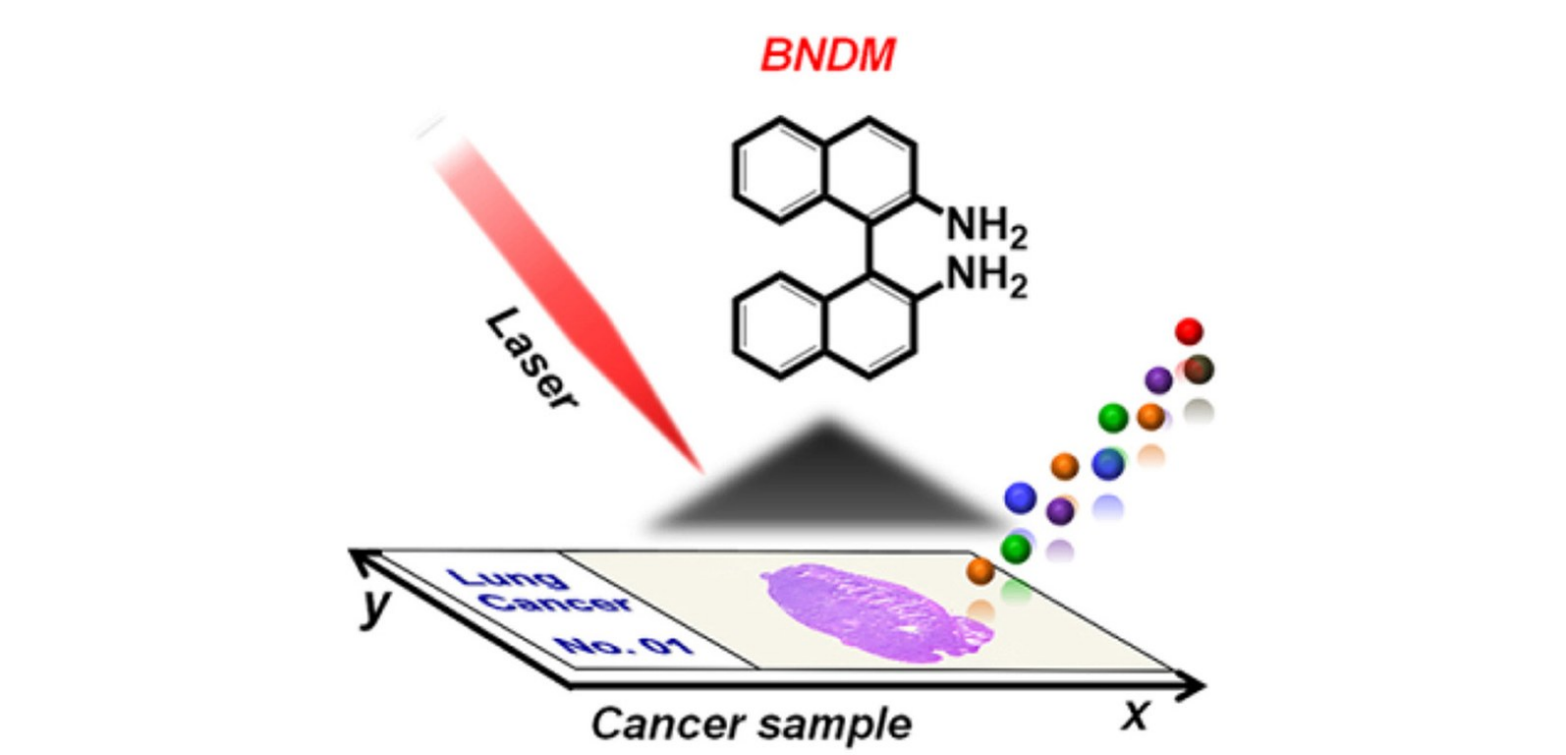
BNDM作为MALDI-MSI的新基质检测代谢物的示意图^[9]^

研究发现非传统基质和基质添加剂可为分析中性脂质提供优势。Dufresne等^[17]^提出了一种用MALDI-MSI检测中性脂质的样品制备方法,使用Na_2_CO_3_或Na_3_PO_4_向组织切片中掺入钠,然后进行2,5-二羟基苯甲酸(DHB)升华。此方法能够检测几种中性脂质,例如胆固醇酯类、羊毛甾醇酯、甘油二酯、脑苷脂和甘油三酯。这种方法已成功应用于脑、肾、肝和肾上腺的MALDI-MSI实验。相较于之前报道的中性脂质的检测方法,这种方法能够以5 μm的空间分辨率使中性脂质可视化。

脂质组中含有较多的碳碳双键位置异构,这给脂质的精确结构分析和定量分析带来挑战。Bednarik等^[11]^利用Paternò-Büchi(PB)反应,使用苯甲醛作为衍生化试剂,将脂质分子中的碳碳双键选择性衍生化,实现了对高不饱和磷脂和糖脂中碳碳双键的定位分析。游离脂肪酸(FFAs)参与信号分子的构成,这些分子对体内的许多代谢活动如脂质和葡萄糖代谢至关重要,但由于其相对分子质量小,在组织中丰度低,电离效率差,给其检测带来困难。Wang等^[12]^使用易于合成的试剂*N*,*N*-二甲基哌嗪碘化物(DMPI)建立了组织原位FFAs衍生化方法,该试剂可以在温和条件下与脂肪酸反应,图2为DMPI与脂肪酸反应的示意图,衍生的FFAs适用于以正离子模式检测。通过使用这种衍生化试剂和已建立的衍生化方法,可以同时检测和成像甲状腺组织中的FFAs和磷脂(PLs),同时提高了检出目标分子的数量和方法灵敏度。

**图 2:**
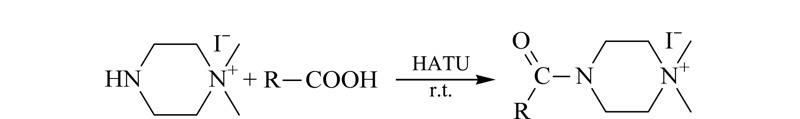
脂肪酸与衍生试剂反应示意图^[12]^

### 1.2 大分子

常见的生物大分子物质包括蛋白质、多糖和核酸等,其相对分子质量从几万到几百万不等,在生物体内存在量变和空间定位的变化,它们在疾病甚至癌症发展过程中发挥重要功能。对大分子进行检测的离子源有MALDI、SIMS、DESI等。Nunez-Naveira等^[18]^用MALDI-TOF MSI对9例肺癌患者呼出气冷凝物(EBC)中的蛋白质分布进行探究,发现EBC中含较高浓度的皮离蛋白和S100A9; Mochiji等^[19]^使用氩气团簇离子源,通过SIMS-MSI检测到了细胞色素C(12327 Da)和糜蛋白酶(25000 Da); Hsu等^[20]^通过nano-DESI检测出质量达到15 kDa的蛋白质。

通过MALDI-MSI检测组织样品中高相对分子质量蛋白质(>25 kDa)分布的一般方法需要先进行原位酶解。原位酶解是将较大的蛋白质水解为多肽,然后对生成的肽段进行原位成像分析,常用的酶为胰蛋白酶^[21]^。组织切片清洗用于去除对测量灵敏度产生不利影响的脂质、盐和其他小分子。清洗方案需要根据目标分析物进行评估选择,表3列举了不同实验目的的洗涤方案。Bastrup等^[25]^将DHB和2-羟基-5-甲氧基苯甲酸混合,并添加磷酸,减少了背景信号的干扰,对阿尔兹海默症病人大脑和淀粉样前体蛋白转基因小鼠的淀粉样蛋白(Aβ)进行MALDI成像,增强了对Aβ蛋白,特别是Aβ1-42的检测效果。Piga等^[26]^以产生的质谱峰数和平均信噪比为标准,评估了不同的洗涤步骤,以及SunChrom自动喷涂机不同的喷涂方式,使用MALDI-FTICR-MSI(matrix-assisted laser desorption/ionization-fourier transform ion cyclotron resonance-mass spectrometry imaging)分析小鼠和人胰腺组织中的完整蛋白,结果表明,在乙醇-水的基础洗涤步骤中加入芥子酸,在喷涂时提高喷嘴的速度可以获得较高质量的图谱。

**表 3:** 大分子成像前的切片洗涤方案

Protocol	Step 1	Step 2	Step 3	Step 4	Step 5	Step 6	Sample	Ref.
1	75% isopropanol 1 min	90% isopropanol 1 min					neurodegenerative disease human skin sample	[22]
2	70% ethanol 2 min	70% ethanol 2 min	100% ethanol 2 min	several washings with cold ACS-grade water	ethanol		animal with multiple skin nodular melanomas	[23]
3	70% ethanol 15s	96% ethanol 15s					multiple sclerosis brain tissue	[24]
4	70% ethanol	99% ethanol	Carnoy’s fluid (ethanol, chloroform, acetic acid)	99.9% ethanol	double-distilled water	99.9% ethanol	APP transgenic mice (APPPS1-21)	[25]
5	70% ethanol 30 s	100% ethanol 30 s	deionized water	70% ethanol 30 s	100% ethanol 30 s		human pancreas tissue sample	[26]

### 1.3 基质喷涂

在MALDI-MSI研究中,常用的基质喷涂方法包括喷枪法、自动喷雾法、升华法。喷枪法属手动方法,受人为因素影响,重现性较差。自动喷雾法可以提供更均匀的基质层,提高了重现性。用升华法喷涂基质可以制备均匀的基质结晶薄层,但是升华法会降低蛋白质的提取效率。Yang等^[27]^发现基质沉积后的重结晶可以提高蛋白质的电离效率。Lin等^[28]^在基质升华和水合作用之后,加入了超声处理,发现*m/z*>10000的蛋白质信号显著增强。通过升华进行的基质沉积一般使用标准玻璃升华器,根据实验要求可以进行改进:Fernandez等^[29]^设计了一种不锈钢升华器,可以更好地控制升华过程中的样品温度和室内真空,使基质沉积的可重复性达到5%。Nakashima等^[30]^将基质均匀地涂覆在胶带上,并通过导电胶带附着在导电载玻片(indium-tin-oxide coated slide, ITO载玻片)上,然后将大鼠脑组织黏附到载玻片上。图3为该方法制备的示意图。与传统方法相比,这种方法可以减少基质沉积的时间和工作量,但是其得到的信号强度较低。

**图 3:**
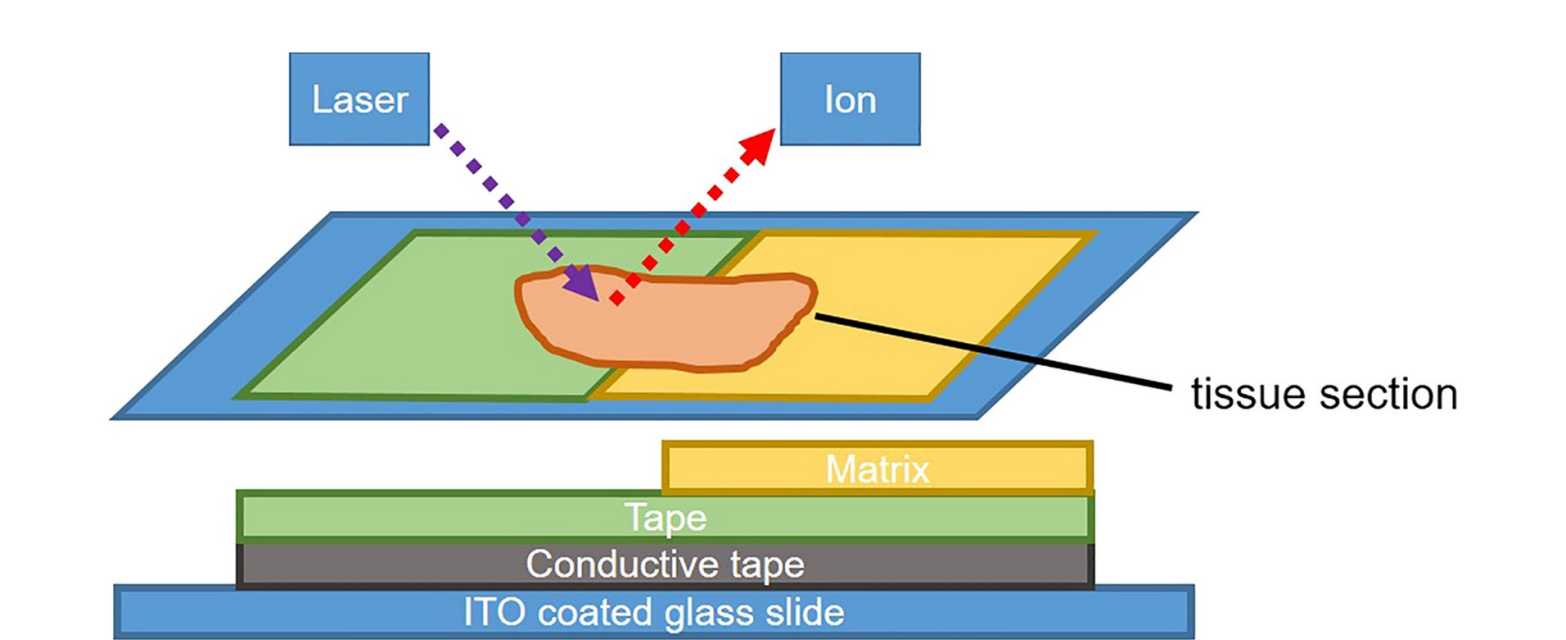
基质沉积方法示意图^[30]^

通过解吸电喷雾电离质谱(DESI-MS)进行蛋白质分析时,灵敏度会随蛋白质质量增加而下降。为了克服此困难,Maser等^[31]^在DESI分析期间,使用润湿笔将预润湿的溶剂(含有乙腈和甲酸)喷涂到样品上,增加了蛋白质溶解时间,改善相对分子质量较大的蛋白质(如牛血清白蛋白)的成像分析。采用该方法可以直接从稀释的山羊血清中检测到白蛋白和相对分子质量更大的蛋白质。

### 1.4 石蜡包埋样品的处理

与新鲜肿瘤样品相比,石蜡包埋组织被广泛应用于人体肿瘤组织的保存,导致了在MSI研究中需要进行脱蜡等前处理。用于MALDI-MSI的福尔马林固定石蜡包埋(formalin-fixed and paraffin-embedded, FFPE)组织切片制备的一般步骤包括:组织分离、固定、脱水、石蜡包埋、切片、脱蜡、清洗、抗原回收、胰蛋白酶消化、基质喷涂^[32]^。图4为对FFPE组织进行MALDI MSI肽分析的工作流程^[33]^。

**图 4:**
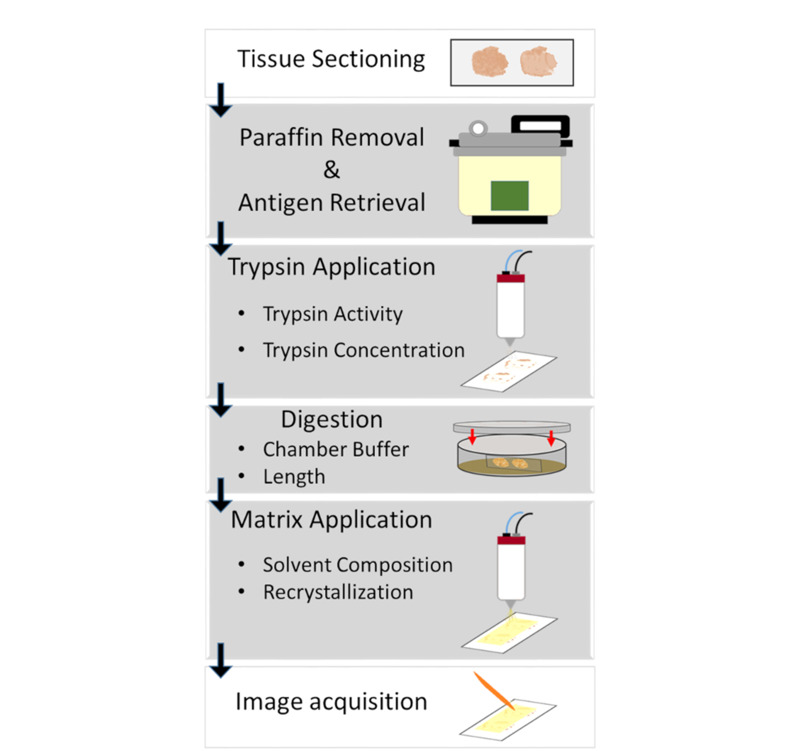
对FFPE组织进行MALDI MSI肽分析的工作流程^[33]^

Hermann等^[34]^优化了溶解胰蛋白酶的溶剂条件、胰蛋白酶的浓度、脱蜡后的清洗过程、胰蛋白酶和基质的喷涂等步骤,用于肾脏、心脏和血管组织的FFPE切片,标准化的操作程序可以提高从不同实验室获得数据的可靠性。

Paine等^[35]^开发了一种能够从FFPE组织中检测内源肽的方法。他们通过MALDI-MSI分析了30年前用福尔马林固定包埋石蜡中的美洲蟑螂的神经内分泌组织,揭示了20多种肽的组织学定位。该方案的样品前处理使用二甲苯和乙醇进行脱蜡处理,省略了清洗、抗原回收以及会干扰MSI对低丰度内源肽检测的酶消化步骤,实现了对内源性肽的高通量质谱成像分析。胶原蛋白和弹性蛋白是所有组织和器官的基本框架,它们的表达和翻译后修饰在生命活动中受到严格的调控。Angel等^[36]^使用胶原酶和基质金属蛋白酶代替常见的胰蛋白酶来消化FFPE组织中的胶原蛋白和弹性蛋白,并使用MALDI-MSI检测消化产物,获取胶原蛋白和弹性蛋白的空间定位。

## 2 质谱成像在乳腺癌中的应用

乳腺癌是女性最常见的恶性肿瘤,发病率在世界范围内呈明显上升趋势^[37]^。因此,建立早期诊断和有效筛查方法能提高乳腺癌患者的生存率。随着分子生物学的发展,质谱成像技术为肿瘤的研究和诊断提供了新的思路,它是一种研究生物组织或细胞中分子组成及分布的新型分析技术,此方法可在无需标记的前提下同时检测肿瘤组织中多种物质的分布。近年来,研究者们为了更好地探索质谱成像在乳腺癌中的应用,主要分为3种模型来进行研究:以乳腺癌细胞为模型的研究,以乳腺癌动物为模型的研究,以临床肿瘤样本为模型的研究。

### 2.1 细胞模型

3D细胞微球是肿瘤研究中不可或缺的体外研究工具,此模型在评估抗肿瘤药物的渗透和代谢方面有很好的应用前景。多项研究成果表明2D和3D细胞体系存在微环境间的差别。Yue等^[38]^利用纳升液相色谱-质谱联用技术对2D和3D结肠癌细胞培养模型的蛋白组进行比较,结果表明细胞在3D培养状态下体内的增殖和扩张速度不如2D培养快,3D培养能更好地反映体内条件。Vidaysky等^[39]^采用液相色谱-质谱联用技术,在2D和3D培养模型中评估乳腺癌细胞的脂质分子代谢情况。研究发现与2D细胞培养相比,3D细胞培养的肿瘤细胞总脂质含量显著下降,酰基甘油与膜脂的比值增加。此外,在恶性程度不同的3D细胞微球体中也存在显著差异。因此,3D细胞体外培养技术更能反映细胞恶性转化和微环境的改变,通过这些脂质分子的空间分布差异可以更好地了解脂质代谢和癌症之间的关系。

Hummon等^[40]^把MALDI-MSI技术应用于HCT116结肠癌多细胞球状体中,他们发现药物对结肠癌多细胞球状体的渗透随时间增加而增加,并且绘制出了药物处理后3种代谢物(SN-38、SN-38葡糖醛酸苷、脱羧代谢物)在细胞球中的定位图。他们还通过组织还原和MALDI-MSI分析,成功地绘制了西妥昔单抗在两种不同结肠癌细胞(HT-29和DLD-1)微球中的时间依赖性渗透和空间分布^[41]^,图5为该实验的机理图。此外,Tucker等^[42]^利用傅里叶变换离子回旋共振(FT-ICR)、MALDI-MSI来探究乳腺癌细胞MCF-7球状体和正常细胞的内源性代谢物差异。他们可以根据代谢物标记来判断这些细胞培养模型的中心区域经历了缺氧加剧或者是氧化应激。

**图 5:**
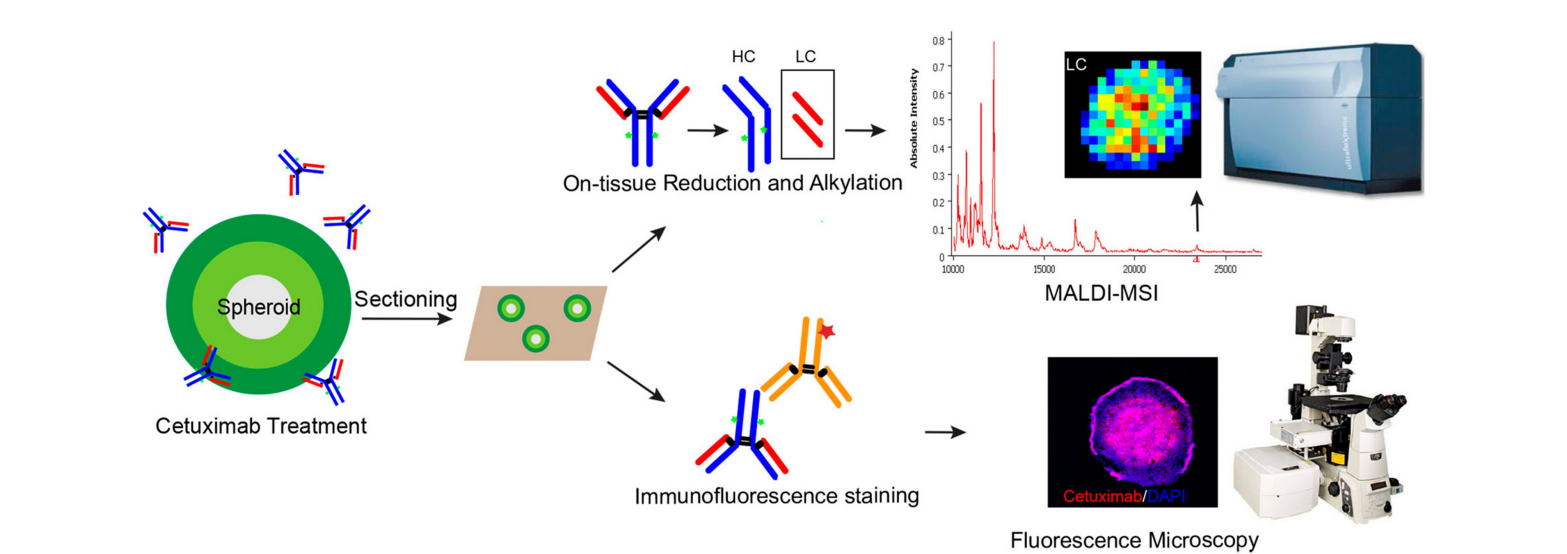
MALDI-MSI检测西妥昔单抗的示意图^[41]^

质谱成像技术在新药开发方面有很大的应用前景。Feist等^[43]^利用多细胞肿瘤球状体(multicellular tumor spheroids, MCTS)的空间异质性来检验药物的分布,通过MALDI-MSI,利用HCT116结肠癌MCTS模型来评价表观遗传药物UNC1999的作用。Theiner等^[44]^用了一种先进的激光灼烧-电感耦合等离子体质谱(LA-ICPMS)装置,用于MCTS的高空间分辨率元素成像,他们测定了人结肠癌细胞HCT116中的磷和铂元素,即此方法可用于筛选金属抗癌药物。表4列举了不同离子源质谱和3D模型在不同癌症中的检测。

**表 4:** 不同离子源质谱、3D模型及其检测内容

Different ion source mass spectrometry	3D model (cancer cells)	Test content	Refs.
NanoLC-MS/MS	HT29	proteomics, phosphorylated proteomics	[38]
LC-MS/MS	MCF10A	lipid metabolism	[39]
MALDI-MSI	HCT 116, HT-29, DLD-1	penetration and distribution of drugs in cancer cells	[40,41]
MALDI-MSI	MCF-7	distribution of adenosine phosphate and glutathione	[42]
MALDI-MSI	HCT116	epigenetic drug UNC1999	[43]
LA-ICPMS	HCT116	phosphorus and platinum in cancer cells	[44]

LA-ICPMS: laser ablation inductively coupled plasma mass spectrometry.

### 2.2 动物模型

肿瘤的发生发展一直是医学领域的重点和难点问题,人类对肿瘤的起因、浸润、转移等方面的分子机理尚不清楚。在肿瘤研究中,建立人肿瘤动物模型是研究肿瘤细胞生长、侵袭和转移的重要手段。因此,合适的肿瘤动物模型对于评价抗癌药物及抗肿瘤免疫治疗的疗效至关重要。为了了解肿瘤的空间异质性,Jiang等^[45]^对来自乳腺肿瘤异种移植的MDA-MB-231-HRE-tdTomato细胞的三维MALDI-MSI数据进行线性判别分析,他们发现低氧调节蛋白在以下几个途径中表达上调,如葡萄糖代谢、肌动蛋白细胞骨架调节、蛋白质折叠和翻译等过程。此外,还发现一些特定的磷脂酰胆碱和鞘磷脂定位在肿瘤的缺氧区,这些脂质和蛋白质可能会转化为缺氧的潜在生物标志物,可能会是潜在的治疗乳腺癌的靶点。

同样,为了了解缺氧带来的肿瘤变化,Mascini等^[46]^以小鼠乳腺癌异种移植为模型,使用MALDI-MSI来研究哌莫硝唑及其代谢,他们证明了哌莫硝唑确实可以作为低氧标记,这些标记在临床诊断和放化疗方面有很大前景。Sun等^[47]^利用MSI方法来研究异种移植小鼠模型肿瘤组织中肉碱的空间分布变化,对17种左旋肉碱进行成像,他们发现参与肉碱系统介导的脂肪酸氧化途径的CPT 1A(carnitine palmitoyltransferase 1A)、CPT 2(carnitine palmitoyltransferase 2)和CRAT(carnitine acetyltransferase)在乳腺癌中异常表达。这也是人们首次发现CPT 2和CRAT在乳腺癌的表达中发生了变化。Reyzer等^[48]^以erbB受体抑制剂OSI-774和Herceptin处理后的HER2转基因鼠为模型,采用MALDI-MSI进行分析,发现当胸腺素β4和泛素下降超过80%时,会出现肿瘤细胞增殖抑制,并诱导细胞凋亡,此效应具有时间和剂量依赖性。此外,他们还预测了OSI-774和Herceptin的协同治疗效果以及耐药作用,表明了基于MALDI-MSI的乳腺癌早期的蛋白质组学变化可用于预测其临床治疗。Johnson等^[49]^以MCF-7乳腺癌异种移植小鼠为模型,采用UPLC-ESI-QTOF-MS(ultra-performance liquid chromatography-electrospray ionization-quadrupole time-of-flight mass spectrometry)对其尿液进行代谢组学分析,发现了酯葡萄糖醛酸、牛磺酸、香豆酸硫酸酯、癸酸葡糖苷酸等5种与乳腺癌相关的代谢物,其在肠道内存在显著性差异表达,这些代谢产物的变化揭示了脂肪酸合成和肠道微生物与抗肿瘤增殖作用之间的相关性。Tanaka等^[50]^用定量质谱成像技术分析了伊帕替尼在小鼠脑转移区的浓度,发现荧光标记的右旋糖苷在脑转移区和脑实质中荧光强度相当,表明血肿瘤屏障保持完整,伊帕替尼有望成为HER2阳性乳腺癌脑转移的治疗药物。

### 2.3 临床肿瘤样本

随着应用分子诊断技术的发展,研究者们根据乳腺癌的基因表达谱不同,将其分为不同亚型^[51]^。目前基于雌激素受体(ER)、孕激素受体(PR)、人类表皮生长因子受体2(HER2)和ki-67等分子受体的表达将乳腺癌分为5种亚型,即管腔A型(luminal A)、管腔B型(luminal B)、HER2阳性型、基底样型/三阴性型以及其他类型。乳腺癌分子分型的研究对乳腺癌的个体治疗和靶向治疗有重要依据。

Kim等^[52]^采用MALDI-MSI鉴定临床样本中的内源性雌激素,并成功检测了芳香酶抑制剂处理的MCF-7细胞系中雌酮代谢表达水平的变化。因此,定量的MALDI-MSI方法有望成为含酮代谢物的靶向代谢组学的通用方法。Rauser等^[53]^利用MSI技术来探究HER2阳性乳腺癌组织中的蛋白标记物,对48例乳腺癌组织进行质谱成像分析,表明HER2状态与特异性多肽/蛋白的表达相关,其中一个富含半胱氨酸的小肠蛋白1(CRIP1)被认为是与HER2过度表达密切相关的蛋白之一,因此,它可以作为判断HER2阳性乳腺癌的特异性的标记物之一。Scott等^[54]^采用MALDI-MSI方法,对人乳腺癌组织相关的N-glycan分布进行评估。他们发现特定的聚糖结构分布在基质区、坏死区和肿瘤区,一系列高甘露糖、分枝糖和岩藻糖基乳糖主要分布在肿瘤区域。此外,在晚期HER2阳性、三阴性和转移性乳腺癌组织中检测到一系列乳糖胺聚糖。这些聚糖的发现为临床的诊疗提供了新的思路,并可作为潜在的预后生物标志物进行进一步的评估。Prentice等^[55]^采用MALDI成像的胰蛋白酶肽来比较10个三阴性乳腺癌肿瘤组织的癌变区域和良性组织区域。通过分析编码这14种蛋白的基因表达与三阴性乳腺癌患者无复发生存率的关系,发现其中9个基因的高表达与较低的无复发生存率相关。相反,在雌激素受体阳性的肿瘤中,这些基因的高表达与这些基因无相关性。该研究为三阴性乳腺癌相关生物标记物的探索提供了新的认识,为乳腺癌的预后和治疗提供了新的方向。

常压敞开式离子化质谱可以在开放环境下对样品进行解吸和离子化,且不需要复杂的样品前处理。Calligaris等^[56]^发现DESI-MSI可以获得具有代谢特征谱图的分子图像,并用来鉴别肿瘤组织和正常组织,有望将DESI-MSI应用在保乳手术中,从而快速检测残余的癌组织。Santoro等^[57]^利用DESI-MSI识别出浸润性乳腺癌(IBC)、原位导管癌(DCIS)和相邻的良性组织(ABT)之间以及乳腺癌分子亚型之间不同的脂质成分。Robison等^[58]^利用DESI-MSI方法来鉴定乳腺癌细胞单层和悬浮培养(转移性)中HER2/p53表达、转移潜能和疾病状态(即癌症与非癌症)的脂质生物标记物。此外,他们还利用DESI-MSI方法来绘制转移性球状体中脂质的空间分布(MDA-MB-231),发现12种脂类与单层细胞培养的转移潜能变化相关,其中3种定位于球形坏死的核心,表明其在营养缺乏的环境中有促进癌细胞存活的潜在作用。在单层MDA-MB-231培养中未检测到的一种脂类物质被定位到球体的外周,这表明它在浸润或增殖中发挥潜在作用。Mao等^[59]^利用AFAI-MSI (air flow assisted ionization-mass spectrometry imaging)通过分析脂质来区分乳腺浸润性导管癌(IDC)和原位乳腺导管癌(DCIS)。他们发现多种亚型和组织结构IDC和DCIS可以用AFAI-MSI进行区分:IDC中磷脂含量高于DCIS,而DCIS中脂肪酸含量高于IDC,标本的分类与组织病理学诊断的吻合度较高,证明此方法有望为临床治疗提供辅助诊断。

## 3 展望

乳腺癌作为女性最常见的肿瘤,受到人们广泛的关注,不同亚型的乳腺癌一般具有不同的病情发展和结局,而如何区分不同的亚型一直以来是临床上需要面对的难题。通过质谱成像技术对乳腺癌进行诊断为其提供了新的研究方向,但目前质谱成像并未广泛应用到临床诊断中,仍然处于起步阶段。在实验过程中,样品制备、仪器控制、数据分析等仍然存在一定的改善空间。此外,采集大量样品,开展大范围的实验,才能为乳腺癌的诊断和治疗带来新的突破。
